# Patient journey and decision processes for anti-amyloid therapy in Alzheimer’s disease

**DOI:** 10.1007/s00415-025-13059-3

**Published:** 2025-04-16

**Authors:** Brant Mittler, Xavier Cambi, Morgan Biskach, Joel Reisman, Ying Wang, Dan Berlowitz, Peter Morin, Donald R. Miller, Karla Brandao-Viruet, Sophie J. Clare, Kevin Z. Xia, Amir Abbas Tahami Monfared, Michael Irizarry, Quanwu Zhang, Weiming Xia

**Affiliations:** 1 UT Health San Antonio, The University of Texas Health Science Center at San Antonio, 7703 Floyd Curl Drive, San Antonio, TX 78229 USA; 2 Department of Pharmacology, Physiology and Biophysics, Boston University Chobanian & Avedisian School of Medicine, 700 Albany Street, Boston, MA 02115 USA; 3 Geriatric Research Education and Clinical Center, Bedford VA Healthcare System, Building 70, Room 202, 200 Springs Road, Bedford, MA 01730 USA; 4 Department of Biological Science, Kennedy College of Sciences, University of Massachusetts Lowell, One University Avenue, Lowell, MA 01854 USA; 5 Center for Healthcare Optimization and Implementation Research, Bedford VA Medical Center, 200 Springs Road, Bedford, MA 01730 USA; 6 Research Service, VA Bedford Healthcare System, 200 Springs Road, Bedford, MA 01730 USA; 7 Wentworth Institute of Technology, 550 Huntington Avenue, Boston, MA 02115 USA; 8 Department of Public Health, Dugan Hall, Zuckerberg College of Health Sciences, University of Massachusetts Lowell, 883 Broadway Street, Lowell, MA 01854 USA; 9 Department of Neurology, Boston University Chobanian & Avedisian School of Medicine, 725 Albany Street, Boston, MA 02118 USA; 10 Center for Population Health, Department of Biomedical and Nutritional Sciences, University of Massachusetts, 710 Pawtucket Street, Lowell, MA 01854 USA; 11 Tufts Medical Center, 800 Washington Street, Boston, MA 02118 USA; 12 Frank H. Netter MD School of Medicine, Quinnipiac University, MNH-338, 370 Bassett Road, North Haven, CT 06473 USA; 13 Alzheimer’s Disease and Brain Health, Eisai Inc., 200 Metro Boulevard, Nutley, NJ 07110 USA; 14 Epidemiology, Biostatistics and Occupational Health, McGill University, 2001 McGill College Avenue, Montreal, QC H3A 1Y7 Canada

**Keywords:** Alzheimer’s disease, Anti-amyloid therapy, Electronic health records, Lecanemab, Mild cognitive impairment

## Abstract

**Introduction:**

We utilized the Veterans Affairs Healthcare System administrative database to study the clinical decision-making processes for anti-amyloid therapy (AAT).

**Methods:**

Patients with clinical notes mentioning lecanemab were identified (March 2023–June 2024) for manual review and structured database queries.

**Results:**

From an initial sample (*N* = 2499), 1064 patients (55,000 notes) were reviewed manually (mean age 76 years; 7.3% women; 9.2% Black; 3.9% Hispanic). The AAT group (*n* = 56) had lower rates of common comorbidities, except post-traumatic stress disorder, than patients excluded from AAT (*n* = 528). The documented notes including “Lack of patient interest/resource constraints” (24.6% vs 3.6%), “anticoagulant use” (23.1% vs 10.7%), and “advanced AD” (18.6% vs 0), supplied partial explanations on exclusion vs inclusion.

**Discussion:**

Only 5.3% of patients reached the point of care of being a candidate, scheduled for, or receiving AAT infusion. Patient preference and clinician discretion, especially regarding modifiable factors (e.g., medication regimens), appreciably influence the patient journey to AAT.

## Background

Alzheimer’s disease is a chronic, progressive, neurodegenerative disorder, typically manifesting clinically after age 65 years, with the risk more than doubling in each subsequent decade of life [1, 2]. Until recently, clinicians could only offer patients palliative treatments to address symptoms [3]. The introduction of anti-amyloid therapy (AAT) has signaled a paradigm shift in Alzheimer’s disease treatment to achieve disease modification [4–6]. Available AATs (lecanemab and donanemab) are indicated for initiation in mild cognitive impairment (MCI) due to Alzheimer’s disease or mild Alzheimer’s dementia (AD) [7–10]; therefore, timely recognition of eligible patients is important.

The Veterans Affairs Healthcare System (VAHS) is the largest integrated healthcare system in the United States (US), encompassing 170 Medical Centers and 1000+ outpatient clinics, and providing care to over 9 million Veterans [11]. Shortly after lecanemab received accelerated pathway approval from the US Food and Drug Administration (FDA) in January 2023 [5], the VAHS issued a lecanemab National Drug Monograph (January 2023) and Criteria for Use (CFU) (February 2023), subsequently announcing coverage for patients meeting the CFU (March 2023) [12, 13]. Lecanemab received traditional FDA approval in July 2023 and the VAHS has since issued 2 CFU updates (August 2023; August 2024 [Appendix, Table 5]) [14–16].

Similar to published Appropriate Use Recommendations (AUR) for lecanemab [17], the VAHS CFU are largely based on the exclusion and inclusion criteria used in Clarity AD, the pivotal phase 3 randomized controlled clinical trial that led to regulatory approval [7]. The key inclusion criteria include fulfilling the diagnosis for MCI due to AD or mild AD, findings indicative of Alzheimer’s disease from specialized testing such as amyloid positron emission tomography (PET) and/or cerebrospinal fluid (CSF) biomarkers, as well as, meeting Alzheimer’s disease cut-offs via specific cognitive studies. Both the VAHS CFU and the AUR are more restrictive than the lecanemab US prescribing information by adding exclusions for anticoagulants, restrictions for seizures in the prior year, and, in the case of the CFU, APOE 4 homozygote status.

While the proportion of potential patients who might qualify for AAT based on clinical trial inclusion and exclusion criteria has been examined [18], there are limited data regarding how the treatment decision process carries through in real world clinical practice [19]. We sought to use the extensive VAHS database, including clinical notes, to examine the patient journey and treatment decision making for AAT.

## Methods

### Database and identification of study sample

All clinical notes from the nationwide VAHS database electronic health records (EHR) were searched to identify patients who may have been considered for the AAT lecanemab from March 13, 2023 to June 16, 2024. The search keywords included “lecanemab”, and “Leqembi”, and potential misspellings. We initially identified a total of 2499 patients with notes containing these keywords. Due to resource limitations and the labor intensiveness of manual notes review, we targeted a sample of 1000 patients (i.e., 40% of the overall sample). A random number generator was used to obtain a uniformly distributed random numbers, enabling random selection of 1019 patients. Since women are underrepresented in the VAHS relative to the general US population, we also chose to include all women from the initially identified patient cohort, leading to the inclusion of an additional 45 women in the final sample of 1064. All clinical notes available for this final sample during our 15-month study window were extracted. Multiple clinical notes per patient could be available for manual review; in all, 55,000 notes were reviewed. Note review was conducted by a physician, a senior epidemiologist, and two doctoral students with physicians’ guidance.

### Patient journey/AAT decision-making categories

Based on review of a preliminary random sample of notes, a 7-point categorization scheme was devised to facilitate description of the AAT patient journey as follows: (1) AAT introduced (first mentioned in a clinical note); (2) Considered a candidate for AAT; (3) AAT scheduled; (4) AAT infused; (5) excluded from AAT; (6) AAT treatment monitoring, and (7) AAT suspended.

Since a single patient could have documentation for multiple categories over time in the 7-point AAT journey categorization scheme, for the purposes of this analysis, the patients were assigned to the category corresponding to the highest step of patient journey that the patient achieved within the reviewed time window of the clinical notes. The patients in categories 2, 3, 4, 6, and 7, were considered the “AAT group”, patients in category 5 were in the “excluded” from ATT group, and those remaining in category 1, were considered as “AAT status not yet determined” during the study window.

### Data parameters and analysis

Information collected during the clinical notes review included clinician types, clinical work-up processes or procedures including brain imaging, CSF biomarker screening, apolipoprotein E (ApoE) testing, and other factors that could impact decision making regarding AAT.

The patient characteristics were extracted from the structured VAHS administrative database and included demographics at the time of the first note mentioning AAT, MCI or AD classified based on case definitions detailed below, and geographic location, healthcare resource utilization, care processes and screening parameters based on VAHS treatment protocols in the year prior to the first AAT note. The structured data were also searched for any use of acetylcholinesterase inhibitors and memantine prior to the first AAT note. Additionally, comorbidities including Charlson Comorbidity Index components [20] as well as health conditions that could impact development of AD were documented in the 2 years prior to the time of the first AAT-related clinical note.

Patients were classified as having MCI or AD based on either (1) having two keyword-identified clinical notes recording MCI or AD in the unstructured EHR (October 2009–February 2024) or (2) having 1 *International Classification of Diseases (ICD)-9-Clinical Modification (CM)* or *ICD-10-CM* diagnostic code for either MCI (*ICD-9-CM* 331.83; *ICD-10-CM* G31.84) or AD (*ICD-9-CM* 331.0; *ICD-10-CM* G30.X) in the structured EHR. Our method for identifying MCI/AD in clinical notes via keywords has been described previously [21]; in the current analysis, the two notes had to be documented 30 to 364 days apart.

Examination of brain imaging, CSF biomarkers, and ApoE in the VAHS database revealed fewer than expected numbers of these procedures and tests, suggesting a need to expand the search into the Centers for Medicare and Medicaid Services (CMS) fee-for-service (FFS) administrative database. Therefore, we examined the VA+CMS data in the year prior to the first AAT note as well as during a 2-year look-back period from June 16, 2024, the end of the study window.

The study findings were summarized for the overall sample and stratified by subgroup classifications. Most data were categorical and reported as number and percentage; continuous parameters (age, outpatient visits) were reported as mean and standard deviation (SD).

## Results

### Patient sample

#### Demographic and clinical characteristics

Demographic and clinical characteristics of the patient sample selected for note review are summarized in Table 1 for all patients (*N* = 1064) as well as for the AAT group (*n* = 56), those excluded from AAT (*n* = 528), and those whose AAT status was not yet determined (*n* = 480). Overall, patients had a mean ± SD age of 76 ± 7.2 years; 7.3% were Female, 9.2% were Black vs 81.5% White, and 3.9% were Hispanic vs 89.8% non-Hispanic. Distribution by US geographic region ranged from 20.6% in the West to 29.3% in the South for the overall group; patients in the AAT group had a markedly higher frequency of residing in the South compared with the other subgroups (46.4% vs 22–35%). Most (73%) patients resided in urban locations, with a similar distribution (72–77% urban) across subgroups.

**Table 1:** Patient demographics and clinical characteristics based on structured VAHS EHR^a^

	All (*N* = 1064)	AAT group^b^ (*n* = 56)	Excluded from AAT (*n* = 528)	AAT status not yet determined (*n* = 480)
Demographics				
Mean±SD age, years	76.2±7.2	75.9±5.3	76.7±7.2	75.7±7.4
Sex, *n* (%)				
Men	986 (92.7)	<60^c^	508 (96.2)	427 (89.0)
Women	78 (7.3)	<10	20 (3.8)	53 (11.0)
Race, *n* (%)				
White	867 (81.5)	47 (83.9)	438 (83.0)	382 (79.6)
Black	98 (9.2)	<10	44 (8.3)	52 (10.8)
Native Hawaiian/Pacific Islander	12 (1.1)	<10	<10	<10
Asian	10 (0.9)	0 (0)	<10	<10
Native American/Alaskan	<10	<10	30 (5.7)	35 (7.3)
Unknown	70 (6.6)	<10	30 (5.7)	35 (7.3)
Ethnicity, *n* (%)				
Hispanic	41 (3.9)	<10	19 (3.6)	<20
Non-Hispanic	956 (89.8)	50 (89.3)	478 (90.5)	428 (89.2)
Unknown	67 (6.3)	<10	31 (5.9)	<40
Geographic region of residence, *n* (%)				
Midwest	253 (23.8)	<10	149 (28.2)	<100
Northeast	280 (26.3)	16 (28.6)	157 (29.7)	107 (22.3)
South	312 (29.3)	26 (46.4)	118 (22.3)	168 (35.0)
West	219 (20.6)	<10	104 (19.7)	<110
Density of residence, *n* (%)				
Rural	285 (26.8)	13 (23.2)	139 (26.3)	133 (27.7)
Urban	779 (73.2)	43 (76.8)	389 (73.7)	347 (72.3)
Clinical characteristics				
AD/MCI/dementia, *n* (%)				
AD^a^	298 (28.0)	18 (32.1)	156 (29.5)	124 (25.8)
MCI^a^	521 (49.0)	27 (48.2)	263 (49.8)	231 (48.1)
Dementia	514 (48.3)	25 (44.6)	288 (54.5)	201 (41.9)
Other Select Comorbidities, *n* (%)				
Hypercholesterolemia	767 (72.1)	34 (60.7)	384 (72.7)	349 (72.7)
Hypertension	744 (69.9)	31 (55.4)	384 (72.7)	329 (68.5)
Obstructive sleep apnea	320 (30.1)	12 (21.4)	169 (32.0)	139 (29.0)
Diabetes	304 (28.6)	11 (19.6)	164 (31.1)	129 (26.9)
PTSD (post-traumatic stress disorder)	229 (21.5)	19 (33.9)	109 (20.6)	101 (21.0)
Obesity	157 (14.8)	<10	74 (14.0)	<80
Cerebrovascular disease	149 (14.0)	<10	80 (15.2)	<70
Kidney disease	139 (13.1)	<10	75 (14.2)	<60
Chronic pulmonary disease	133 (12.5)	<10	63 (11.9)	<70
Cancer	132 (12.4)	<10	70 (13.3)	<60
Peripheral vascular disease	110 (10.3)	<10	64 (12.1)	<50
Chronic heart failure	75 (7.0)	<10	44 (8.3)	<40
Liver disease	44 (4.1)	<10	24 (4.5)	<20
Myocardial infarction	34 (3.2)	<10	20 (3.8)	<20
Traumatic brain injury	33 (3.1)	0 (0)	15 (2.8)	<20
Rheumatic disease	18 (1.7)	<10	11 (2.1)	<10
Peptic ulcer	<10	0 (0.0)	<10	<10
Hemiplegia or paraplegia	<10	<10	<10	<10
Human immunodeficiency virus	<10	0 (0.0)	<10	<10

^a^Data in this table were derived from the administrative EHR, in most cases from the structured data (i.e., diagnostic coding); however, classification of a patient as having MCI or AD could be based on having either a relevant diagnosis code for MCI (*ICD-9-CM* 331.83, *ICD-10-CM* G31.84) or for AD (*ICD-9-CM* 331.0, *ICD-10-CM* G30.X) in structured data or having 2 keyword identified clinical notes documenting either MCI/AD in unstructured data, the notes being 30 to 364 days apart. In the overall study sample, the AAT group, those excluded from AAT, and those whose status was not yet determined, 245/1064 (23.0%), 11/56 (19.6%), 109/528 (20.6%), and 125/480 (26.0%) of patients, respectively were not classified as either AD or MCI using our case definitions

^b^Categories 2, 3, 4, 6, 7

^c^Values in certain cells cannot be reported directly due to VAHS data suppression rules regarding reporting *n* values of 1–10 from the structured database; some cells with values >10 require suppression if presenting their value(s) would enable inference, via arithmetic, of the data in a suppressed cell

*AD* Alzheimer’s disease, *AAT* anti-amyloid therapy, *EHR* electronic health record, *MCI* mild cognitive impairment, *VAHS* Veterans Affairs Healthcare System

We were able to classify 28% of patients as MCI and 49% with AD using our case definition based on unstructured clinical notes and/or ICD coding in the structured data (Table 1). Additionally, 48.3% of patients had a non-specific dementia diagnostic code. Any use of acetylcholinesterase inhibitors and/or memantine was found in 56% of patients overall: 48.2% in the AAT group, 57.2% among those excluded from AAT, and 55.6% of patients with AAT status not yet determined (Appendix, Table 6).

Hypercholesterolemia and hypertension were the most common comorbidities, occurring in 72.1% and 69.9% of all patients, respectively (Table 1). Other common comorbidities occurring in over 20% of the overall sample included obstructive sleep apnea, diabetes, and post-traumatic stress disorder. The rates of the most common comorbidities were generally lower among patients in the AAT group, except for post-traumatic stress disorder, which was present in 33.9% of patients in the AAT group compared with approximately 21% of patients in the other subgroups.

#### Additional structured database findings

Additional information from the structured administrative database reflecting VAHS healthcare utilization and care processes in the year prior to the first AAT-related note is summarized in the appendix (Table 7). All patients had at least 1 outpatient visit and 10% experienced a hospitalization within the VAHS in the year prior to the first AAT note. Most patients (98.9%) had a primary care visit in the VAHS, whereas 57.2%, 43.8%, and 34.3% had specialty care visits with mental health, neurology, and geriatrics, respectively. The frequency of specialty visits was lower in the AAT group: mental health 42.9%, neurology 41.1%, and geriatrics 25.0%, compared to 58.7%, 49.1%, and 40.3%, respectively in the excluded patient group. About 90% of patients had at least 1 mental health-, cognition-, and/or function-related assessment, with comparable frequency across the subgroups. Magnetic resonance imaging (MRI) was the most common brain imaging method in the VAHS structured database, found in 24.6% of patients overall, followed by brain CT (16.7%), and brain PET (2.9%). CSF biomarker and ApoE testing rates in the VAHS structured database were each identified in <2% of patients.

Further review of brain imaging, biomarker, and ApoE data from the VA+CMS structured administrative databases in the year prior to the first AAT-related note (Appendix, Table 8A) identified brain MRI, brain CT, brain PET, CSF biomarkers, and ApoE in 30.9%, 23.0%, 3.4%, 2.5%, and 1.1%, of all patients, respectively; MRI rates were higher in the AAT vs excluded groups (44.6% vs 30.7%).

Among other potential factors that could impact decision making regarding AAT, psychiatric/behavioral conditions were found in 46.8% of patients overall in the VAHS structured data, and comparable across subgroups (Table 7). Substance abuse and suicidal ideation, referenced in the VAHS CFU, were found in 2.3% and <1.0% of patients, respectively, with no cases in the AAT group. Comorbidities that may potentially predispose a patient to brain hemorrhage such as brain aneurysm, cerebral amyloid angiopathy (CAA), nontraumatic intracerebral hemorrhage (ICH), and nontraumatic subarachnoid hemorrhage (SAH) were each found in <1% of patients overall, with no cases in the AAT group. Use of aspirin, anticoagulants, other antiplatelet drugs, and antipsychotics was found in 16.0%, 14.2%, 6.2%, and 6.4% of all patients, respectively.

### Patient journey with AAT

#### Distribution of patients by AAT journey category

**Fig. 1:**
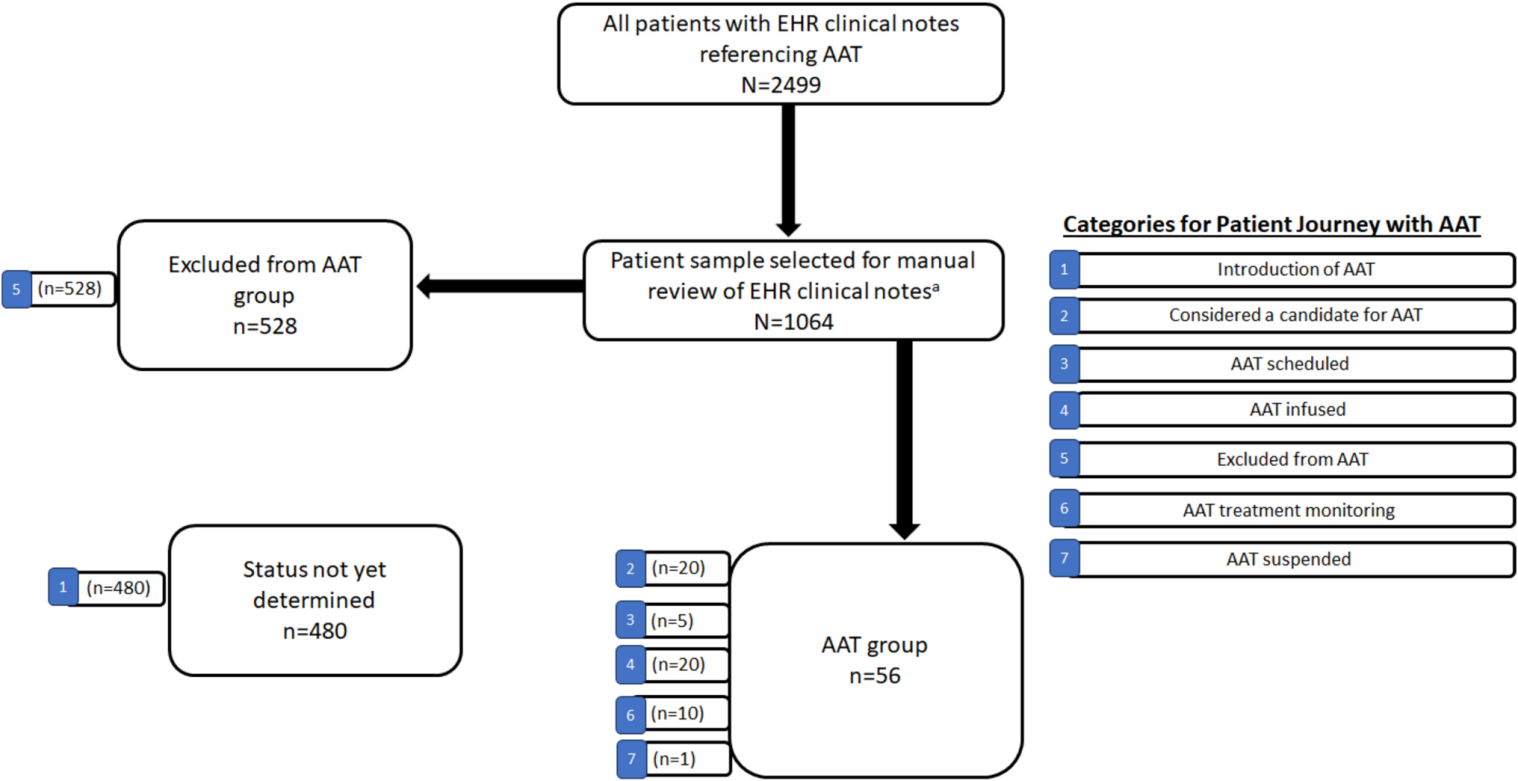
Categorization by the latest step in patient journey with AAT. ^a^1019 patients were randomly selected from the initial sample of 2499 patients with notes referencing AAT; any women who had not been selected (*n* = 45) were added to enrich their representation to produce a final sample of 1064 patients

Based on note review, the patients were categorized into their latest step in the AAT clinical decision process in the VAHS (Fig. 1). During the 15-month study window, 5.3% (56/1064) of patients were classified in the AAT group based on either being considered a candidate for AAT (category 2, *n* = 20), being scheduled for AAT (category 3, *n* = 5), AAT infused (category 4, *n* = 20), AAT treatment monitoring (category 6, *n* = 10), or AAT suspended (category 7, *n* = 1) (Fig. 1). Approximately half (49.6%; 528/1064) of patients in the final sample were excluded from AAT (category 5). The remaining patients (45.1%; 480/1064) were in an early segment of the patient journey to AAT during the study window (i.e., category 1, “introduction of AAT”); in 60/480 of these “category 1” cases, AAT was referenced in the clinical notes, but did not appear to have been discussed with the patient.

#### Distribution of healthcare provider types

In the overall study sample (*N* = 1064), note review during the 15-month study window found that patients most frequently had notes by neurologists (53.3%), followed by primary care providers (PCPs; 43.8%), geriatric psychiatrists (28.5%), nurses (23.5%), and geriatricians (16.7%) (Table 2). Compared with the overall sample and other subgroups, more patients in the AAT group had notes from primary care providers (76.8%) and geriatric psychiatrists (35.7%), but fewer had notes from neurologists (39.3%).

**Table 2:** Proportion of patients with notes by provider type found during the AAT patient journey observation period^a,b^

Provider type, *n* (%)	All patients *N* = 1064	AAT group (*n* = 56)	Excluded from AAT (*n* = 528)	AAT status not yet determined (*n* = 480)
Neurologist	567 (53.3)	22 (39.3)	292 (55.3)	253 (52.7)
Primary care	466 (43.8)	43 (76.8)	153 (29.0)	270 (56.3)
Geriatric psychiatrist	303 (28.5)	20 (35.7)	125 (23.7)	158 (32.9)
Nurse	250 (23.5)	16 (28.6)	103 (19.5)	131 (27.3)
Geriatrician	178 (16.7)	9 (16.1)	95 (18.0)	74 (15.4)

^a^Based on manual review of clinical notes

^b^Each patient could have notes from multiple provider types

*AAT* anti-amyloid therapy

#### Brain imaging, CSF biomarkers, and genetic testing

Neuroimaging and AD-related laboratory testing found during note review over the 15-month study window is summarized in Table 3. Patients in the AAT group had markedly higher rates of amyloid PET (35.7%) compared to the overall sample and other subgroups (15–16%). Likewise, the AAT group had higher rates of testing for ApoE (28.6%) and CSF biomarkers (12.5%) than other groups (each <6%).

**Table 3:** Brain imaging and Alzheimer’s-related testing found during the AAT patient journey observation period^a^

Testing, *n* (%)	All patients *N* = 1064	AAT group (*n* = 56)	Excluded from AAT (*n* = 528)	AAT status not yet determined (*n* = 480)
Brain imaging				
MRI	528 (49.6)	30 (53.6)	242 (45.8)	256 (53.3)
CT	255 (24.0)	7 (12.5)	106 (20.1)	142 (29.6)
Amyloid PET	167 (15.7)	20 (35.7)	77 (14.6)	70 (14.6)
FDG-PET	89 (8.4)	4 (7.1)	51 (9.7)	34 (7.1)
ApoE	62 (5.8)	16 (28.6)	26 (4.9)	20 (4.2)
CSF biomarkers	48 (4.5)	7 (12.5)	24 (4.5)	17 (3.5)

^a^Based on manual review of clinical notes

*AAT* anti-amyloid therapy, *ApoE* apolipoprotein E, *CSF* cerebrospinal fluid, *CT* computed tomography, *FDG* fludeoxyglucose-18, *MRI* magnetic resonance imaging, *PET* positron emission tomography

Further review of brain imaging, biomarker, and ApoE data from the VA+CMS structured administrative databases (from end of study, looking back 2 years; Appendix, Table 8B) found rates of MRI, CT, and brain PET were 47.2%, 38.3%, and 5.3%, respectively for all patients; combined rates of brain MRI and/or brain CT were higher in the AAT vs excluded groups (75% vs 66.5%) due to higher MRI rates in the AAT group (71.4% vs 46.6%). Overall 9.1% of patients had brain PET or CSF biomarker testing within the VA+CMS structured data, with generally comparable rates across subgroups. ApoE testing in the VA+CMS data was found in 7.6% of patients overall, with markedly higher rates in the AAT vs excluded groups (32.1% vs 6.1%).

#### Other decision factors

Note review captured the mention of factors that could impact treatment decisions with AAT (Table 4). Psychiatric/behavioral conditions were common in the overall sample (47.4%) and more frequent in the AAT group (51.8%) vs the excluded group (43.0%). The rates of substance abuse and suicidal ideation in the overall sample were 5.0% and 1.1%, respectively, with higher rates in excluded vs AAT groups: 6.3% vs 1.8% for substance abuse; 1.1% vs no cases for suicidal ideation.

**Table 4:** AAT screening factors found during the patient journey observation period^a^

	All patients *N* = 1064	AAT group (*n* = 56)	Excluded from AAT (*n* = 528)	AAT status not yet determined (*n* = 480)
Comorbidities related to mental health, *n* (%)				
Psychiatric/behavioral conditions	504 (47.4)	29 (51.8)	227 (43.0)	248 (51.7)
Substance abuse	53 (5.0)	1 (1.8)	33 (6.3)	19 (4.0)
Suicidal ideation	12 (1.1)	0 (0)	6 (1.1)	6 (1.3)
Comorbidities related to brain hemorrhage, *n* (%)				
Brain aneurysm	20 (1.9)	1 (1.8)	7 (1.3)	12 (2.5)
CAA	20 (1.9)	0 (0)	7 (1.3)	13 (2.7)
ICH	17 (1.6)	0 (0)	5 (0.9)	12 (2.5)
SAH	9 (0.8)	0 (0)	5 (0.9)	4 (0.8)
Select medications, *n* (%)				
Aspirin	418 (39.3)	32 (57.1)	165 (31.3)	221 (46.0)
Anti-coagulants	194 (18.2)	6 (10.7)	122 (23.1)	66 (13.8)
Anti-platelet Drugs	90 (8.5)	5 (8.9)	39 (7.4)	46 (9.6)
Anti-psychotics	21 (2.0)	1 (1.8)	10 (1.9)	10 (2.1)
Other clinical factors, *n* (%)				
Advanced AD	114 (10.7)	0 (0)	98 (18.6)	16 (3.3)
Pacemaker^b^	82 (7.7)	4 (7.1)	32 (6.1)	46 (9.6)
Other comorbidiites^c^	73 (6.9)	3 (5.4)	56 (10.6)	14 (2.9)
Age <65 years old	44 (4.1)	1 (1.8)	25 (4.7)	18 (3.8)
Negative amyloid PET	31 (2.9)	0 (0)	14 (2.7)	17 (3.5)
Preclinical Alzheimer’s	15 (1.4)	1 (1.8)	7 (1.3)	7 (1.5)
ApoE 4/4^d^	10 (0.9)	1 (1.8)	6 (1.1)	3 (0.6)
Healthcare system/patient factors, *n* (%)				
Limited availability of AAT/system constraints^e^	190 (17.9)	13 (23.2)	102 (19.3)	75 (15.6)
Lack of patient interest or resource constraints	138 (13.0)	2 (3.6)	130 (24.6)	6 (1.3)
Potential risk of adverse events (risk-benefit)	66 (6.2)	2 (3.6)	52 (9.8)	12 (2.5)
Deliberation about initiating AAT	60 (5.6)	6 (10.7)	19 (3.6)	35 (7.3)
Transportation or commuting barriers	32 (3.0)	1 (1.8)	26 (4.9)	5 (1.0)

All data are presented as *n* (%)

^a^Based on manual review of clinical notes

^b^The newer generation of MRI compatible pacemakers may explain the presence in 7% of patients in the AAT group

^c^This category encompassed additional comorbidities that were documented in the notes, including cancer, traumatic brain injury/head trauma, Parkinson’s disease, seizures, etc

^d^Homozygous for ApoE *ε4* allele

^e^Some VAHS medical centers did not offer AAT during the study window.

*AAT* anti-amyloid therapy, *AD* Alzheimer’s dementia, *ApoE* apolipoprotein E, *CAA* cerebral amyloid angiopathy, *CFU* criteria for use, *ICH* intracerebral hemorrhage, *SAH* subarachnoid hemorrhage, *VAHS* Veterans Affairs Healthcare System

In the overall sample, note review found risk factors for brain hemorrhage such as brain aneurysm, CAA, nontraumatic ICH, and nontraumatic SAH in 1.9%, 1.9%, 1.6%, and 0.8% of patients, with no cases of CAA, ICH, or SAH in the AAT groups (Table 4). Nonspecific neurologic events such as brain microbleed(s) and stroke were mentioned in 17% of patients overall.

Mention of aspirin was found in 39.3% of patients during note review, and was more common in the AAT vs other groups (57.1% vs 31.3–46.0%) (Table 4). Mention of other antiplatelet drugs was comparable across groups (approximately 7–10%); whereas anticoagulants were found in 18.2% of all patients, and approximately twice as common in the excluded vs AAT groups (23.1% vs 10.7%).

No patients in the AAT group had advanced AD vs 18.6% of excluded patients (Table 4). Pacemakers were found in 7.1% of patients overall and did not differ notably between the AAT and excluded groups. Additional comorbidities that were documented in the notes, such as cancer and traumatic brain injury, were also more frequently mentioned in excluded vs AAT groups (10.6% vs 5.4%). Negative amyloid PET was found in 2.9% of patients, but no patients in the AAT group. Among healthcare system factors that could impact decision-making, the most common in the overall sample (17.9%) was limited availability of AAT/system constraints, with a slightly higher rate in the AAT vs excluded group (23.2% vs 19.3%) (Table 4). The lack of patient interest or personal constraints, including logistical barriers related to the need for biweekly visits for AAT, was noted in 13% of the sample overall, with markedly higher rates in excluded patients vs the AAT group (24.6% vs 3.6%). Discussion of potential risk of adverse events (risk-benefit) were not highly prevalent in the notes, but were more commonly mentioned in excluded vs AAT groups (9.8% vs 3.6%) as were transportation or commuting barriers (4.9% vs 1.8%). Of note, deliberation about commitment to AAT was more commonly documented among patients who received AAT than those who were excluded (10.7% vs 3.6%).

## Discussion

This study offers unique insights into the AAT treatment decision-making process in the VAHS, the largest integrated healthcare system in the US, from both the clinician and patient perspectives. In our sample of 1064 patients who had at least 1 clinical note mentioning AAT/lecanemab, 5.3% were classified in the AAT group during the 15-month study window. Patients in the AAT group generally had a lower comorbidity burden than those excluded from AAT, apart from PTSD. A lower rate of specialty visits in the AAT group was observed, and may be due to the lower comorbidity burden. Nonetheless, most patients across groups received cognitive, functional, or mental health assessments, suggesting due diligence in the Alzheimer’s clinical workup prior to AAT-related discussions. Note review revealed substantially higher proportions of patients in the AAT group had amyloid PET or CSF biomarker testing than found in structured data, suggesting the possibility that biomarker testing procedures may have been performed outside of the VAHS or CMS FFS databases.

The proportion of patients classified in the AAT group in our study is consistent with recent reports [18, 19]. In a cross-sectional sample of 237 Mayo Clinic Study of Aging (MCSA) participants who were classified as having possible or probable Alzheimer’s disease, 8% were found eligible for lecanemab based on application of the Clarity AD clinical trial inclusion/exclusion criteria [18]. The findings from the MCSA sample are interesting, but do not capture real-world details regarding the actual decision-making process. A retrospective review from a memory center in Kentucky reported that 6% (71/1168) of patients with primary ICD-10 diagnostic coding for either AD, MCI, memory loss and/or dementia were selected for treatment with lecanemab [19]. Several reasons for exclusion reported in the memory center study were also identified in our note review, including advanced dementia, patient concerns about adverse events/risks, burden of bi-weekly visits, and transportation/commuting-related concerns [19]. The memory center study also cited cost among reasons patients decline lecanemab. Another recent report identified denial of coverage by private insurers/payors among the major barriers to use of AAT [22]. A unique aspect of the VAHS is that, at the individual patient level, cost and insurance authorization are not typically adjudicated at the point of service; nonetheless, lecanemab is a non-formulary drug and the VAHS CFU must be met to obtain approval.

Consistent with prior reports in Veterans with dementia, including AD, our patient sample had a high rate of psychiatric comorbidity [23, 24]. Since the VAHS CFU states that patients should not receive lecanemab if they have a “mental health condition that may be a contributing/primary cause of cognitive impairment”, such conditions are key considerations in decision-making. Psychiatric/behavioral conditions were prevalent in the AAT group, suggesting that clinicians are using their discretion regarding the potential contribution of these comorbidities to cognitive impairment. While structured data from the year prior to first AAT note showed that overall rates of psychiatric/behavioral conditions was comparable in AAT vs excluded groups, PTSD was higher in the ATT group. Additionally, the note review found that behavioral/psychiatric conditions were more frequently mentioned in the AAT vs excluded groups. We speculate that increased chart notation could possibly reflect targeted screening for these conditions according to the CFU. It is also possible that motivation to try a new treatment was higher among patients with certain mental health conditions.

Anticoagulant/antiplatelet medications were common in our overall patient sample. While use of these drug classes is not contraindicated according to the FDA approved prescribing information for lecanemab [9], these classes are listed as reasons for exclusion in the VAHS CFU (Appendix, Table 5) [16]. Published AUR for lecanemab recommend against its concomitant use with anticoagulants, but allow for use with standard doses of aspirin or other antiplatelet medications [17]. The most recent VAHS CFU (August 2024) [16] also clarify that aspirin is an exception. Mention of aspirin was found during manual chart review in over half of patients in the AAT group; we speculate that many of these cases likely reflected low-dose (81 mg/day) regimens. In the overall sample, the rate of aspirin use was notably lower in the structured data (16.0%) in the year prior to the first AAT note compared with the rate found in note review (39.3%), with the latter likely reflecting documentation of over-the-counter use. Of interest, a Komodo Research Database study found anticoagulant use in 3.7% of patients during lecanemab treatment [25]. We also found anticoagulant use in the AAT group, both via note review and in the structured database. Collectively, these findings underscore that clinician judgment has an important role in decision making regarding AAT [26].

The Komodo database study reported that 93% of the patients received infusions in the urban setting [25]. In our sample, approximately one-fourth of patients overall resided in rural areas; however, there was no notable disparity based on rurality in the AAT group vs those excluded from AAT.

Another important observation in our investigation is that about two-fifths of patients overall had notes by PCPs. This has potential implications for the treatment decision-making process since, within the VAHS, prescription of AAT is restricted to select VAHS-based specialist types. Undoubtedly, PCPs will have an important role in patient identification and referral for AAT. It is imperative that healthcare systems assure adequate educational and administrative support regarding AAT in PCP settings.

Although our data did not show a disparity between AAT and other groups based on rurality, this may be a consideration since elderly Veterans with dementia residing in rural areas [27], may have limited access to specialist care; telehealth may be one means to bridge such care gaps [28–31].

### Limitations

The clinical notes review offers detailed insights into the patient journey whereas structured real world data are typically lacking documentation of such clinical information as reasons for treatment exclusions, patient’s interest in treatment, and care delivery factors. The VAHS has one of the most comprehensive, sophisticated, and searchable EHRs in the world; however, despite the rich degree of detail and ease of use, it was challenging to extract all potentially relevant information related to the treatment decision-making process. For example, even with manual review of clinical notes, the details of patient-provider discussion regarding AAT risk-benefit profiles were often not discernable. Additionally, amyloid load and levels of other biomarkers were not readily found within the clinical notes, and may require expanded search into neuroradiology reports. We were unable to identify reasons for all patients who chose not to seek treatment with AAT within the study window due to lack of specific information in the clinical notes. Additionally, note review was conducted by multiple individuals, thus there is potential for some variability in data collection and categorization.

### Conclusions

This study evaluated multiple real world data sources including clinical notes from various healthcare provider specialties and structured administrative data from the VAHS and CMS. We attempted to reconstruct the patient journey to AAT with a focus on clinical treatment decision processes from a multifaceted perspective. Only 5.3% of patients in our sample eventually reached the point of care where they were either considered a candidate, scheduled for, or receiving AAT infusion. Advanced AD, anticoagulant use and substance abuse were more frequently noted in the excluded vs AAT groups. Lack of patient interest or resource constraints were also more frequently noted in the excluded group, whereas deliberation regarding AAT was more frequently noted in the AAT group, suggesting that patient choice plays an important role in decision making for treatment with AAT. Both patient preference and clinician discretion, especially regarding modifiable factors such as medication regimens, appreciably influence decisions regarding AAT. Overall, enhanced patient-clinician communications on the benefits and risks of AAT are likely to support more timely treatment decision making and adoption of AAT by eligible patients.

## Figures and Tables

**Table 5:**
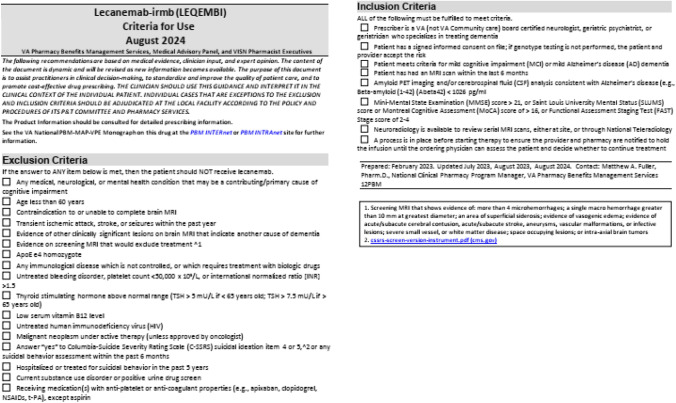
VAHS CFU, August 2024

https://www.va.gov/formularyadvisor/DOC_PDF/CFU_Lecanemab-irmb_LEQEMBI_CFU_Aug_2024.pdf

*CFU* criteria for use, *VAHS* Veterans Affairs Healthcare System

## Data Availability

Request for accessing available data will be submitted for approval by the Department of Veterans Affairs. All data are fully de-identified before access.

## Appendix

See Tables 5, 6, 7, and 8.

**Table 6:** Any use of acetylcholinesterase inhibitors and/or memantine in the VAHS Structured Administrative Database Prior to the First AAT-related Note

	All (*N* = 1064)	AAT group (*n* = 56)	Excluded from AAT (*n* = 528)	AAT status not yet determined (*n* = 480)
Any use of AChEI and/or memantine, *n* (%)	596 (56.0)	27 (48.2)	302 (57.2)	267 (55.6)
Any use of an AChEI^a^, *n* (%)				
Donepezil	497 (46.7)	23 (41.1)	255 (48.3)	219 (45.6)
Galantamine	55 (5.2)	0 (0)	33 (6.3)	22 (4.6)
Rivastigmine	60 (5.6)	<10	36 (6.8)	<30
Tacrine	<10	0 (0)	<10	<10
Any use of memantine, *n* (%)	219 (20.6)	12 (21.4)	109 (20.6)	98 (20.4)

^a^Values cannot be reported directly due to VAHS data suppression rules regarding reporting n values of 1 to 10 from the structured database; some cells with values >10 require suppression if presenting their value(s) would enable inference, via arithmetic, of the data in a suppressed cell

*AAT* anti-amyloid therapy, *AChEI* acetylcholinesterase inhibitors**,** *VAHS* Veterans Affairs Healthcare System

**Table 7:** Healthcare Utilization, Care Processes, and Other Select Parameters/Screening Factors in the Structured VAHS Administrative Database Within the 1-year Period Prior to the First AAT-related Note^**a**^

	All (*N* = 1064)	AAT group (*n* = 56)	Excluded from AAT (*n* = 528)	AAT status not yet determined (*n* = 480)
Outpatient visits, *n* (%)^b^				
Mean (SD)	44.1 (33.4)	30.8 (27.8)	46.5 (35.5)	42.9 (31.2)
0 visits	0 (0)	0 (0)	0 (0)	0 (0)
1–9 visits	73 (6.9)	11 (19.6)	28 (5.3)	34 (7.1)
10–49	622 (58.5)	33 (58.9)	308 (58.3)	281 (58.5)
50+	369 (34.7)	12 (21.4)	192 (36.4)	165 (34.4)
Any hospitalization, *n* (%)	106 (10.0)	<10	57 (12.1)	<50
Outpatient visits by specialty, *n* (%)				
Primary care, any visit	1052 (98.9)	54 (96.4)	525 (99.4)	473 (98.5)
Mental health services, any visit	609 (57.2)	24 (42.9)	310 (58.7)	275 (57.3)
Neurology, any visit	466 (43.8)	23 (41.1)	259 (49.1)	184 (38.3)
Geriatrics, any visit	365 (34.3)	14 (25.0)	213 (40.3)	138 (28.8)
Any use of cognitive, functional, and/or mental health assessment or screening tools, *n* (%)	957 (89.9)	49 (87.5)	483 (91.5)	425 (88.5)
Brain imaging, *n* (%)				
MRI	262 (24.6)	9 (16.1)	137 (25.9)	116 (24.2)
CT	178 (16.7)	<10	96 (18.2)	<80
MRI and/or CT	376 (35.3)	11 (19.6)	201 (38.1)	164 (34.2)
PET^d^	31 (2.9)	<10	18 (3.4)	<20
CSF biomarkers, *n* (%)	19 (1.8)	0 (0)	<20	<10
CSF biomarkers and/or brain PET^d^, *n* (%)	48 (4.5)	<10	27 (5.1)	<20
ApoE, *n* (%)	<10	0 (0)	<10	<10
Comorbidities related to mental health, *n* (%)				
Psychiatric/behavioral conditions	498 (46.8)	26 (46.4)	257 (48.7)	215 (44.8)
Substance abuse	24 (2.3)	0 (0)	<20	<10
Suicidal ideation	<10	0 (0)	<10	<10
Comorbidities related to brain hemorrhage, n (%)				
Brain aneurysm	<10	0 (0)	0 (0)	<10
CAA	<10	0 (0)	<10	<10
Nontraumatic ICH	<10	0 (0)	<10	<10
Nontraumatic SAH	0 (0)	0 (0)	0 (0)	0 (0)
Select medications, *n* (%)				
Aspirin	170 (16.0)	<10	77 (14.6)	<90
Anti-coagulants	151 (14.2)	<10	109 (20.6)	<40
Anti-platelet drugs	66 (6.2)	<10	36 (6.8)	<30
Anti-psychotics	68 (6.4)	<10	39 (7.4)	<30

^a^Defined as 1–365 days prior to first AAT-related note

^b^Multiple visits in a single day were counted as 1 visit

^c^Values cannot be reported directly due to VAHS data suppression rules regarding reporting n values of 1 to 10 from the structured database; some cells with values >10 require suppression if presenting their value(s) would enable inference, via arithmetic, of the data in a suppressed cell

^d^Amyloid vs FDG PET could not be discerned from structured data

*AAT* anti-amyloid therapy, *ApoE* apolipoprotein E, *CAA* cerebral amyloid angiopathy, *CSF* cerebrospinal fluid, *CT* computed tomography, *ICH* intracerebral hemorrhage, *MRI* magnetic resonance imaging, *PET* positron emission tomography, *SAH* subarachnoid hemorrhage, *VAHS* Veterans Affairs Healthcare System

**Table 8:** Testing in VA+CMS Structured Administrative Database (A) Within the 1-year Period Prior to the First AAT-related Note^**a**^; (B) Within the 2-year Period Prior to the Last Day of the Study Window

A				
Testing, *n* (%)	All (*N* = 1064)	AAT group (*n* = 56)	Excluded from AAT (*n* = 528)	AAT status not yet determined (*n* = 480)
Brain imaging				
MRI	329 (30.9)	25 (44.6)	162 (30.7)	142 (29.6)
CT	245 (23.0)	<10	133 (25.2)	<110
MRI and/or CT	476 (44.7)	27 (48.2)	247 (46.8)	202 (42.1)
PET^b^	36 (3.4)	<10	23 (4.4)	<20
CSF biomarkers	27 (2.5)	<10	13 (2.5)	<20
CSF biomarkers and/or brain PET^b^	60 (5.6)	<10	34 (6.4)	<30
ApoE	12 (1.1)	<10	<10	<10

B				
Testing, *n* (%)	All (*N* = 1064)	AAT group (*n* = 56)	Excluded from AAT (*n* = 528)	AAT status not yet determined (*n* = 480)
Brain Imaging				
MRI	502 (47.2)	40 (71.4)	246 (46.6)	216 (45.0)
CT	407 (38.3)	14 (25.0)	212 (40.2)	181 (37.7)
MRI and/or CT	693 (65.1)	42 (75.0)	351 (66.5)	300 (62.5)
PET^b^	56 (5.3)	<10	32 (6.1)	<30
CSF biomarkers	47 (4.4)	<10	23 (4.4)	<30
CSF biomarkers and/or brain PET^b^	97 (9.1)	<10	50 (9.7)	<50
ApoE	81 (7.6)	18 (32.1)	32 (6.1)	31 (6.5)

^a^Defined as 1–365 days prior to first AAT-related note

^b^Amyloid vs FDG PET could not be discerned from structured data

^c^Values cannot be reported directly due to CMS data suppression rules regarding reporting n values of 1 to 10 from the structured database as delineated in: US Department of Health & Human Services. Guidance Protocol. CMS Cell Suppression Policy. January 1, 2020. Available at: https://www.hhs.gov/guidance/document/cms-cell-suppression-policy Accessed February 16, 2025. Some cells with values >10 require suppression if presenting their value(s) would enable inference, via arithmetic, of the data in a suppressed cell

*AAT* anti-amyloid therapy, *ApoE* apolipoprotein E, *CMS* Center for Medicare and Medicaid Services, *CSF* cerebrospinal fluid, *CT* computed tomography, *MRI* magnetic resonance imaging, *PET* positron emission tomography
